# Received

**Published:** 1868-04

**Authors:** 


Received.—We have to acknowledge the receipt of bottles of California Brandy, Port Wine and Angelica Wine, from Perkins, Stern & Co., 14 and 16 Vesey street, New York, and 103 Tremont street, Boston. The specimens received are very fine indeed, so far as we are able to judge; they seem to us perfect in all respects except one—the BOTTLES are a great deal TOO SMALL.

We have also received a very beautiful and valuable box of the finest maple sugar we have ever seen, with the following note, which explains to others all we are permitted to know ourselves:

*Sappytown, April* 13, 1868.

To the Editor of the Buffalo Medical and Surgical Journal:

My Dear Doctor:—Please find enclosed "Sugar Pills" for old school physicians, to be taken with hot cakes or waffles, warranted free from calomel or any deleterious drug, purely vegetable, pleasant to take; children cry for them; they correct acidity of the stomach and disposition; the Mahomedan Life Insurance Company issues a free policy to every person who eats these pills during the month of April. Please give them a trial and send for more if you like them. They may be, and probably are, a little sappy, but don't pronounce them an "immeasurable swindle."

Yours recuperatively and homœopathically, F. A. C.

